# Boosting Biocatalytic Efficiency: Engineering of Chitinase Chit33 with Chitin and Cellulose Binding Domains for Sustainable Chitin Conversion

**DOI:** 10.1021/acs.jafc.4c10364

**Published:** 2025-04-25

**Authors:** María Martínez-Ranz, Peter E. Kidibule, Elena Jiménez-Ortega, Jesús Valcárcel, José Antonio Vázquez, Julia Sanz-Aparicio, María Fernández-Lobato

**Affiliations:** † Department of Molecular Biology, Centre of Molecular Biology Severo Ochoa, CSIC-UAM, University Autonomous of Madrid, Madrid 28049, Spain; ‡ Faculty of Chemistry, Biotechnology, and Food Sciences, Norwegian University of Life Sciences, P.O. Box 5003, Elizabeth N-1432 Ås, Norway; § Department of Crystallography and Structural Biology, Institute of Physical Chemistry Blas Cabrera, CSIC, Madrid 28006, Spain; ∥ Recycling and Valorisation of Waste Materials Group (REVAL), Institute of Marine Research, IIM-CSIC, Galicia 36208, Spain

**Keywords:** Chitinase Chit33, chitooligosaccharides, carbohydrate binding domains, specificity, chitin beads, cellulose beads

## Abstract

Endochitinase Chit33 has shown great potential in converting chitin, a recalcitrant waste, into bioactive chitooligosaccharides (COS). This study evaluates how cellulose-binding domain (CBD) and chitin-binding domain (ChBD) affect the hydrolytic activity and product specificity of Chit33. Recombinant proteins were produced and isolated with a simple yeast extracellular medium concentration. The domain functionality was proved using chitin and cellulose supports. ChBD provided more stable immobilization than CBD but reduced the Chit33 activity. CBD enhanced the enzyme activity on both colloidal (α-/β-allomorphs) and crystalline chitin, doubling it on α-chitin, although not on their deacetylated forms. Besides, CBD increased the COS production from the colloidal forms of α-/β-chitin (by 30% and 85%, respectively) and expanded the product diversity from 1 to 9 *N*-acetylglucosamine units. In contrast, Chit33-ChBD predominantly yielded chitin tetrasaccharides. These findings highlight the importance of selecting appropriate binding domains to tailor product specificity, as polymerization and acetylation degrees directly impact the COS biological properties.

## Graphical abstract

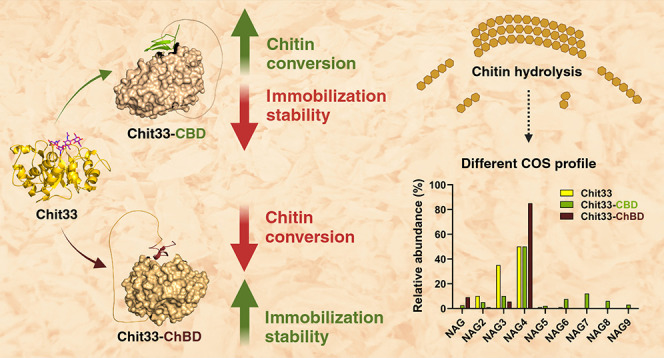

## 1 Introduction

Chitin, the most abundant nitrogen-containing polymer in nature, is a basic component of the hard parts of organisms, such as lobsters, shrimps, insects, and molluscs, as well as fungal cell walls, among many others. This nonwater-soluble polysaccharide is composed of β-(1–4) linked *N*-acetylglucosamine (NAG) units and in nature is packed in highly ordered nanofibrous structures. Chitin crystalline nanofibers occur in different allomorphs, which differ in the packing and polarities of adjacent polymeric NAG chains: α-chitin, in which chains are arranged with an antiparallel packing strongly maintained by hydrogen bonds, and that is mainly found in crustacean shells, fungal cell walls, and insect cuticles; β-chitin, where chains are arranged in parallel, spaced further apart, which makes the hydrogen bonds weaker (hence, more accessible to hydrolytic enzymes), mainly extracted from squid pens and also present in some individuals of the phylum Annelida; and last, γ-chitin, a mixture of α- and β-chitin, structurally very close to α-chitin (even considered as a variant of α-chitin), which is much less frequently found in nature, the cocoon of the Orgyia dubia moth being an example.^1–3^ Chitin deacetylation produces chitosan, which consists of NAG and d-glucosamine (GlcN) units, the latter normally constituting more than 55% of the polymer. Both chitin and chitosan show inherent properties such as biocompatibility, biodegradability, nontoxicity, and nonimmunogenicity, which give them a wide range of industrial applications. However, the poor aqueous solubility at neutral pH values of chitin and chitosan limits their use, which makes their hydrolysis products, chitin and chitosan oligomers (chitooligosaccharides; COS), gain biotechnological interest in food, health, cosmetic, and agriculture fields because they retain key properties of the native polymers. Among many other functions, in health, COS exhibit anti-inflammatory, antioxidant, and antimicrobial effects, modulate the immune system, and maintain intestinal health. In agriculture, they activate plant defense mechanisms and elicit plant immunity to control diseases, and in the food industry, their antimicrobial properties help extend food shelf life.^4–7^

Chitinases are glycosyl hydrolases (GH) widely distributed in all kingdoms of life. They display different functions, including structural modification in chitin-containing organisms, transformation of insoluble chitin into easily digestible nutrients, or protection against pathogens.^3,7^ In general, the chitinases are divided into two groups depending on the mode of action: *endo*-chitinases that hydrolyze internal glycosidic bonds of chitin randomly and *exo*-chitinases that cleave the β-(1–4) bonds from the polymer ends.^8^ In addition, based on their structural determinants and catalytic mechanism, chitinolytic enzymes are included in the carbohydrate active enzymes database (CAZy; http://www.cazy.org) in the three GH families 18, 19, and 20, with GH18 being the most studied one.^9^

The simplest chitinases contain only one catalytic domain, but they can also include secondary modules such as carbohydrate-binding modules (CBMs), fibronectin type III (FnIII), and immunoglobulin-like ones, among others, which specifically recognize and bind biological compounds (CAZy)­.^10,11^ The CBMs are mainly located at the N-terminal end of plant chitinases and either at the C- or N-terminal ends of the bacterial or fungal members. They are classified into three main types based on their substrate interactions: type A binds to crystalline polysaccharides such as cellulose and chitin via planar, aromatic-rich surfaces; type B interacts with internal glycan chains through extended clefts that accommodate longer oligosaccharides; and type C recognizes exposed or short-chain carbohydrates.^12,13^ CBMs are also structurally classified into families from 1 to 106 (CAZy) and are usually connected to the protein catalytic domain through peptide linkers, which appears to influence the flexibility and binding capacity of the binding domains.^14^ CBMs can increase the enzymes catalytic efficiency, and their elimination provokes an activity decline on long polysaccharides, as the CBM family 5 (CBM5)-type A fused to the *endo*-cellulase CeIE from Acetivibrio thermocellus.^15^ In addition, chitinases fused to CBMs (originally chitin or cellulose binding domains) have shown improved catalytic efficiency, thermostability, or even a change in substrate specificity, as in chitinases ChiB and ChiC from Serratia marcescens fused to CBM5 and CBM family 12 (CBM12)-type A, respectively.^16–19^ These domains have also been used to immobilize many different enzymes to allow recycling of the biocatalyst in numerous industrial processes, thereby reducing its cost and enabling easier product separation.^20,21^

The *endo*-chitinase Chit33 from Trichoderma harzianum is a 321 amino acid residue protein (including a putative signal peptide of 19 residues), which has already been expressed in Pichia pastoris. Activity testing of this enzyme against different substrates indicates that chitin tetrasaccharide (NAG_4_) is the main hydrolysis product obtained from colloidal chitin (CC). The three-dimensional structure of Chit33 has also been solved, and it features a shallow catalytic tunnel with exposed substrate-binding grooves.^4,22^ Here, we report the production of two chimeric variants of Chit33 fused to the chitin-binding domains (ChBD) of the chitinase ChiA from Nicotiana tabacum, a CBM family 18 (CBM18)-type C, and the CBD of the cellobiohydrolase II (CBHII) from Trichoderma reesei, a CBM family 1 (CBM1)-type A. The functionality of both protein variants was demonstrated by analyzing their ability to bind to cellulose and chitin supports as well as their utility for chitinolytic conversion of different substrates into COS, thereby adding value to chitin-rich waste through the production of biologically active compounds. Putative folding of the two chimeras was also analyzed using AlphaFold.

## 2 Materials and Methods

### 2.1 Substrates

Chitin substrates with different degrees of deacetylation (DD) and polymerization (DP) were used in this work. Chitin (coarse flakes, DD ≤ 8%) from shrimp shells was used for the preparation of colloidal chitin (CC or α-CC), as previously reported.^23^ Briefly, 175 mL of 10 M HCl, including 10 g of chitin, was maintained at 4 °C for 16 h and then mixed with 1 L of ethanol; the chitin floccules were precipitated after 16 h, collected by centrifugation at 5000×*g* for 10 min, and finally washed with distilled water until the solution reached pH 4.5. Beta-colloidal chitin (β-CC) was obtained in a similar way from squid pen flakes (Glentham Life Sciences; Corsham, UK). α-Chitin powder and *N*-acetyl-glucosamine (NAG) were from Sigma-Aldrich (Madrid, Spain). Chitosan CHIT100 (100–300 kDa; 92% DD) and CHIT600 (600–800 kDa; 90% DD) from shrimp shells were from Acros Organics (Geel, Belgium); chitosan from squid pen Q2 (268 kDa; 89% DD), Q3 (147 kDa; 89% DD), and Q4 (339 kDa; 82% DD) were isolated and characterized, as previously described.^24^ Details of the isolation process are shown in Figure S1. All other reagents were of the highest purity grade possible.

### 2.2 Microorganisms, Growth, and Protein Expression Conditions

Escherichia coli *DH5*α was used as the host for DNA manipulations following the standard methods. Ampicillin (100 μg/mL) was used as the transformant selection media. P. pastoris GS115 (*his4*-; Invitrogen, Carlsbad, CA, USA; also, Komagataella phaffii) was used as an expression host and was cultured in YEP (1% yeast extract, 2% peptone, 2% dextrose; all w/v) at 30 °C and 250 rpm shaking. Yeast transformants were selected on MD (Yeast Nitrogen Base 1.34%, glucose 2%, and biotin 4 × 10^–5^%; all w/v) agar plates. For heterologous protein production, yeast transformants were first grown at 30 °C during 24 h in BMG (same as MD but potassium phosphate 100 mM pH 6.0 and glycerol 1% instead of glucose) and then transferred to BMM (same as BMG but methanol 0.5% (v/v) instead of glycerol), being supplemented with 0.5% methanol every 24 h for the protein expression. Growth was monitored spectrophotometrically at 600 nm (OD_600_).

### 2.3 Constructions of the Chit33 Chimeras

Constructions pIB4-CHIT33-CBD and pIB4-CHIT33-ChBD, including gene *chit33* (X80006.1; 909 bp, with the TAA stop codon and without the initial 57 bp encoding a 19-residue signal peptide) fused to the CBD of the gene *cellobiohydrolase II* from T. reesei (GU724763.1; 36-residue peptide; positions 26–62 in ADC83999.1) and the ChBD of chitinase ChiA from N. tabacum (X16939; 41-residue peptide; positions 24–65 in P08252), were both obtained using a restriction-free cloning strategy.^25^ Plasmid pGEX4T-2-CHIT33-CBD included sequences codifying the CBD (108 bp) and the linker CBHII (138 bp encoding a 46-residue peptide; positions 62–108 in ADC83999.1) of the cellobiohydrolase II fused to the initial 174 bp of *chit33*.^26^ Plasmid pGEX-5X-2-CHIT33-ChBD included sequences codifying the ChBD (123 bp) of the chitinase ChiA linked also to the linker CBHII and the first 174 bp of *chit33*.^27^ Plasmid pIB4-CHIT33, a derivative of pIB4 (*His4*), included the 909 bp gene *chit33* fused to the Saccharomyces cerevisiae MFα1 secretion signal. Expression of the heterologous proteins was subject to the control of the methanol-regulated alcohol oxidase promoter (*AOX1p*).^4^ Both CBMs had been successfully used previously with *exo*-chitinase Chit42 from T. harzianum.^27^ Briefly, pGEX4T-2-CHIT33-CBD was used as a template to obtain the cassette *cbd-chit33* using primers pIB4CHIT33-CBDF: 5′-tctcgagaaaagagaggctgaagctTGCTCAAGCGTCTGGG-3′ (in lowercase the sequence complementary to the MFα of the pIB4 derivative plasmid and in uppercase the starting sequences of the *cbd*) and pIB4CHIT33R: 5′- actgaggaacagtcatgtctaagaagcttTTACCTCAAAGCATTGACAACCT-3′ (in lowercase the sequence complementary to the insertion site in the pIB4 derivative plasmid and in uppercase the end of gene *chit33*). Similarly, pGEX4T-2-CHIT33-ChBD was used as a template for ChBD amplification using primers pIB4CHIT33-ChBDF: 5′-tctcgagaaaagagaggctgaagctCTAGCACAATGTGGTTCCCAG-3′ (in lowercase the sequence complementary to the MFα included in the pIB4 derivative vector and in uppercase the initial part of the *chbd* sequence) and pIB4CHIT33R. Phusion High-fidelity DNA polymerase (NEB, Ipswich, UK) was used with the following conditions: (i) 98 °C for 30 s; (ii) 25 cycles of 98 °C for 10 s, 56 °C for 30 s and 72 °C for 15 s; and (iii) final extension at 72 °C for 300 s. The PCR products of 474 bp (*cbd-*5′*chit33* fusion) and 489 bp (*chbd*-5′*chit33* fusion) were purified from agarose gel using the NZYgelpure kit (NZYTech, Lisbon, Portugal) and used as a primer in a second PCR reaction, where plasmid pIB4-CHIT33 was the template using the same conditions of PCR as above but with 220 s of extension in (ii) instead of 15 s. The product of this PCR was treated with *Dpn*I for 2 h to degrade the methylated template DNA and directly used to transform E. coli. Positive colonies, including constructions pIB4-CHIT33-CBD and pIB4-CHIT33-ChBD, were detected by PCR using the AOX primers AOX1F: 5′-GACTGGTTCCAATTGACAAGC-3′ and AOX1R: 5′-GCAAATGGCATTCTGACATCC-3′, which flank the *cbd*-*chit33* or *chbd*-*chit33* cassettes and were verified by sequencing.

### 2.4 Pichia pastoris Transformation, Protein Expression, and Concentration

Constructions pIB4-CHIT33-CBD and pIB4-CHIT33-ChBD were linearized with *Stu*I (into *His4*) and transformed into P. pastoris by electroporation according to the manual for protein expression in P. pastoris (Invitrogen, Carlsbad, CA, USA). Integration of the chimeric protein in the yeast transformants was confirmed by PCR using the above-mentioned AOX primers. Transformants, including the empty vector pIB4, were also obtained and used as controls. Expression of chitinases in P. pastoris was analyzed using 180 mL of BMM medium cultivated during 120 h in 1 L flask, and the heterologous chitinase activity was evaluated in culture filtrates. Cells were removed by centrifugation at 6000×*g* for 15 min, and extracellular fractions were filtered through glass microfiber filters GF/C (Whatman, Maidstone, UK) and 0.45–0.22 μm pore membranes (Millipore, Burlington, MA, USA) and stored at −70 °C. When needed, the proteins were concentrated through 30,000 Da MWCO PES membranes by using a Vivaflow 50 system (Sartorius, Gottingen, Germany). Protein expression was analyzed in sodium dodecyl sulfate-polyacrylamide gel electrophoresis (SDS-PAGE 12%) using InstantBlue protein stain (Expedeon, Cambridge, UK) and the Precision Plus Protein Standards 10–250 kDa (Bio-Rad, CA, USA) markers. Proteins were directly quantified from the SDS-PAGE gel bands.

### 2.5 Chitinase Activity and Kinetic Analysis

Unless otherwise indicated, chitinase activity was determined spectrophotometrically by evaluating the amount of reducing sugar obtained from chitinous materials. Standard reaction mixtures included 400 μL of 1% (w/v) CC in 0.1 M potassium phosphate, pH 6.0, and up to 100 μL of the enzymatic solution to be evaluated. The reaction was incubated in a Thermo Shaker TS-100 (Boeco, Hamburg, Germany) at 45 °C and 900 rpm during 1 h. Reactions were boiled for 10 min and then centrifuged (12000×*g*; 5 min). The amount of reducing sugars in the supernatants was quantified using the 3,5-dinitrosalicylic acid (DNS; at 540 nm) method in a 96-well microplate, as previously described,^4,23^ with NAG 0–3 mg/mL as the calibration curve. All reactions were performed in triplicate. One unit of chitinase activity (U) was defined as that corresponding to the release of 1 μmol of reducing sugars per minute. For estimation of the chitinase activity at different temperature values in the range of 30–70 °C, 0.1 M sodium acetate pH 5.0 was used, and for estimation of pH dependence of this activity, 0.1 M sodium citrate or potassium phosphate for the pH range 3–5.5 or 5.5–8, respectively, were used. The kinetic constants were determined with GraphPad Prism software using 0.2–15 mg/mL CC in sodium acetate at pH 5.0 for Chit33 and 4.5 for the chimeric variants. All of the reactions were incubated at the enzyme’s optimum temperature.

### 2.6 Chitin Conversion and Soluble Products Analysis

In the chitin conversion assays, reactions (500 μL-final volume) including CC or chitin powder (8 mg/mL) and 7.9 μg of the protein variant showing chitinase activity, all in sodium acetate 0.1 M pH 5 (for the wild-type: wt Chit33) or pH 4.5 (for the chimeric variants), were maintained at the optimum temperature for each enzyme variant (45 °C for Chit33, 50 °C for Chit33-CBD and 55 °C for Chit33-ChBD) during 24 h. The nonhydrolyzed substrate was precipitated by centrifugation (12,000×*g*; 20 min), lyophilized, dried in an incubator for 24 h at 110 °C, and weighed. Control reactions were carried out using the same conditions but with enzymes previously inactivated by boiling for 10 min. The percentage of chitin conversion into COS (also referred to as % of chitin solubilization) was estimated by comparing the weight of the precipitates obtained from reactions mediated by the active protein variants to those from the control reactions. The molecular mass of COS was assessed by MALDI-TOF-MS using a mass spectrometer with Ultraflex III TOF/TOF (Bruker, Billerica, MA, USA) and NdYAC laser.^4^ Mass spectra (MS) were obtained in the positive reflector mode within the mass interval 40–5000 Da, with external calibration and with 20 mg/mL 2,5-dihydroxybenzoic in acetonitrile (3:7; v/v) as a matrix. Samples were mixed with the matrix in a 4:1 proportion and 0.5 μL were analyzed. The matrix and sample were cocrystallized on the probe by allowing the solvent to evaporate at room temperature.

### 2.7 Synthesis and Characterization of Cellulose and Chitin Beads

Chitin beads were produced, as previously referred^28^ with some modifications. Briefly, chitin flakes were dissolved in *N*–*N*-dimethylacetamide with 5% LiCl during 5 h with agitation to obtain a chitin solution of 0.4% (w/v). Then, the solution was filtered through a strainer with fine holes (<1 mm). To form the chitin beads, the filtered chitin solution was dripped (19 G needle; about 1 μL) on 500 mL of ethanol 96% using a Cole Parmer 4052 Syringe Pump at 1 mL/min. A similar procedure was used to produce cellulose beads by dropping cellulose acetate (20%, w/v, in DMSO) into 0.5 M HCl and magnetically stirring. Then, the cellulose beads were incubated for 1 h in 1 M NaOH for saponification of the acetate, as reported before.^29,30^ Both types of beads were collected, then washed with distilled water, and stored at 4 °C. For the scanning electron microscopy (SEM) analysis, the cellulose and chitin beads were previously dried at 37 °C for 16 h and coated with gold under a vacuum prior to the SEM microscopic observation. Images were obtained using an FE-SEM S-4700 cold cathode field emission microscope (Hitachi, Japan) at the Instituto de Cerámica y Vidrio (CSIC, Madrid).

### 2.8 Binding Assay and Reaction Cycles

In this assay, 40 mg of wet cellulose or 30 mg of wet chitin beads per tube were incubated in binding buffer (BB; 200 mM NaCl, 0.1 M sodium phosphate pH 5.5) with 10.0 U of Chit33-CBD or 7.0 U for Chit33-ChBD using a revolver rotator (Labnet Inc., USA) at 4 °C. After 16 h of incubation at pH 5, the BB was removed together with the unbound enzyme by pipetting. The biocatalysts (enzyme-bead complexes) were washed with 0.5 mL of first distilled water and then 50 mM sodium phosphate pH 5.5 by pipetting-resuspension, mixed with 0.4 mL of CC (8 mg/mL at pH 4.5, 0.1 M sodium acetate), and incubated at 55 °C for 30 min to evaluate its chitinase activity. Chitinase activity of all liquid fractions was estimated as referred to above, assessing the reducing sugars using DNS after inactivation of the enzyme solution by 10 min of boiling followed by centrifugation. For the reuse of the biocatalysts analysis, 5-reaction cycles were performed, and the solubilization of CC was estimated, as for free enzymes, as indicated above. An overview of the general process followed in the immobilization of biocatalysts and the reaction cycles is shown in Figure S2.

### 2.9 Software and Structure Predictions

Proteins were quantified from SDS-PAGE using ImageJ 1.53 K (National Institutes of Health, NIH). In the kinetic analysis, plotting and data analysis of the obtained curves were carried out using GraphPad Prism software (version 10.0), fitting data to a nonlinear regression based on the Michaelis–Menten equation. The structure of the chimeric proteins was predicted by using AlphaFold2/ColabFold,^31^ generating five models for each sequence. Mean pLDDT (predicted local distance difference test) over the structure was used for ranking five generated models, all of them giving a high overall quality, as evaluated by the server (mean pLDDT 85–86).

## 3 Results

### 3.1 Obtention of the Chimeric Chit33 Variants and Their Heterologous Expression

To explore the effect of CBM domains on the catalytic function of the *endo*-chitinase Chit33, two chimeric variants of this protein were generated from constructions pIB4-CHIT33-CBD and pIB4-CHIT33-ChBD. In both chimeras, the signal peptide (19 residues) of Chit33 was removed, and the CBM was connected through the Ser/Pro-rich linker CBHII from T. reesei. The entire construct was preceded by the MFα1 secretion signal (Figure 1A). In addition, sequences codifying for the two chimeras were flanked by the *AOX1* promoter and terminator sequences, and consequently, their expression was induced by methanol. The P. pastoris transformants expressing the two chimeric proteins showed extracellular chitinolytic activity on α-CC, with maximum activity levels of about 110 and 43.2 mU/mL when including CBD and ChBD, respectively (Figure 1B). Protein bands of about 50 kDa were also detected in SDS-PAGE (Figure 1C,D), with a maximum production of about 41 and 24 mg/L (96 h cultures; 2.7 and 0.81 U/mg, respectively).

**Figure 1:**
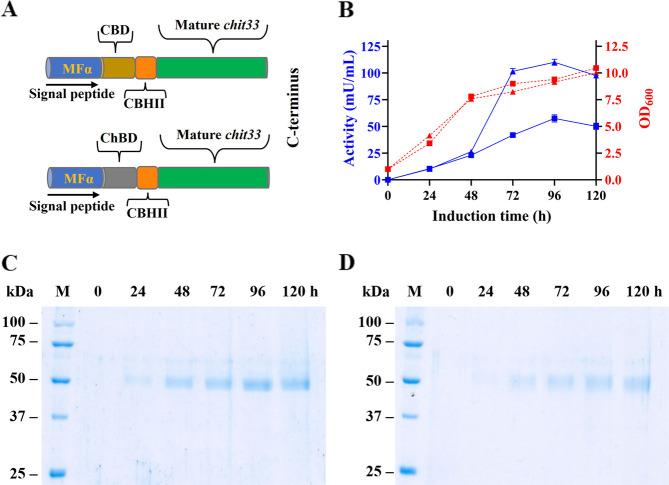
Constructs and expression profiles of yeast cultures expressing the two chimeric variants of Chit33. (A) Scheme of the chimeras assembled. CBHII means CBHII linker. (B) P. pastoris transformants, including constructions pIB4-CHIT33-CBD (triangles) and pIB4-CHIT33-ChBD (squares), were cultured in BMM. Cell growths (red, dashed lines) and extracellular chitinase activity (blue, solid lines) were evaluated using CC as the substrate at 45 °C pH 6, as previously described for the Chit33 wt. Each point of activity represents the average of three independent measurements, and standard errors are indicated. SDS-PAGE analysis of the chimeric variants (C) Chit33-CBD and (D) Chit33-ChBD expressed in P. pastoris. Extracellular yeast media (16 μL) were evaluated during the referred methanol induction times. Numbers on the left of each panel indicate the positions of molecular mass standards (lane M).

### 3.2 Enzymatic Properties of the Chit33 Chimeric Variants

The fusion of the two CBMs used in this work caused considerable changes in the hydrolytic activity of Chit33 on CC (α-CC; Table 1). Thus, the variants including CBD and ChBD exhibited maximum activity at 50 and 55 °C, respectively (Figure S3). Both variants maintained over 80% of their activity (here considered as optimal activity) at temperatures slightly higher (about 11–22%) from those previously reported for the wild-type (wt) enzyme (45 °C and activity ≥80% in the 40–50 °C range).^4^ The two chimeric proteins also showed maximum activity at pH 4.5 (Figure S3) and retained at least 80% of their activity at pH values slightly lower (about 10%) than those of the wt enzyme (pH 5.0; activity ≥80% at pH 4.5–5.5).^4^ In addition, fusion of CBD to Chit33 improved both its specific activity on CC, by almost 90%, and its apparent catalytic efficiency (defined by the *k*_cat_/*K*_m_ ratio) by 54%, basically due to an apparent *K*_m_ decrease (Table 1 and Figure S4). However, the fusion of ChBD to Chit33 reduced its specific activity by almost 35% and its apparent catalytic efficiency by 77%, basically due to an apparent *K*_m_ increase. The specific activity decrease of chimeric proteins has been previously reported and associated with the length of the linker used between the CBM and the catalytic β-sheet barrel, as well as whether the CBM is fused to the C- or N-terminal of the proteins.^32^ Therefore, the fusion of the two used CBMs to Chit33 not only increased the optimal temperature of the enzyme-mediated reaction but also substantially modified its apparent catalytic efficiency, and while ChBD clearly reduced it (by more than 75%), CBD enhanced it (by almost 55%), thus increasing the potential of Chit33 for COS production.

**Table 1:** Enzymatic Characteristics and Apparent Kinetic Constants of Referred Chit33 Variants^a^

variant	opt. *T* (° C)	opt. pH	spec. act. (U/mg)	*K*_m_ (mg/mL)	*k*_cat_ (s^–1^)	*k*_cat_/*K*_m_ (mL/mg s)
Chit33 wt	40–50	5.0–6.0	2.17 ± 0.02	4.6 ± 0.7	6.2 ± 0.08	1.3 ± 0.2
Chit33-CBD	47–55	3.5–4.8	4.13 ± 0.14	0.8 ± 0.2	1.6 ± 0.06	2.0 ± 0.2
Chit33-ChBD	50–60	4.2–4.8	1.42 ± 0.02	16.9 ± 5.2	5.0 ± 0.2	0.3 ± 0.2

a Colloidal chitin (α-CC) was used as a substrate. Opt. *T* and pH mean temperature and the pH range of activity ≥80% of the maximum value, respectively. Spec. act., specific activity. Each protein variant was evaluated at their maximum activity conditions (Chit33 wt, 45 °C pH 5; Chit33-CBD 50 °C, Chit33-ChBD 55 °C, and both pH 4.5). Data are the average of three independent experiments, and standard errors are indicated. The values of *k*_cat_ were calculated from *V*_max_ considering a Chit33, Chit33-CBD, and Chit33-ChBD protein molecular masses of 33, 43.5, and 44.4 kDa, respectively; wt, wild-type.

### 3.3 Union of the Chimeric Variants to Cellulose and Chitin Supports

The functionality of the two chimeric proteins formed in this work was analyzed by evaluating their capacity to bind to the obtained cellulose and chitin beads. Cellulose and chitin beads were about 2.5 and 1 mm in the wet form and 2 and 0.5 mm in the dry form, respectively (measured using millimeter paper; Figure S5). Dry bead size was also analyzed by SEM with similar results (Figure 2A). The immobilized biocatalysts, containing Chit33-CBD and Chit33-ChBD, retained about 75% (7.5 U) and 21.4% (1.5 U) of the chitinolytic activity used to form them, respectively. As expected, Chit33-CBD and Chit33-ChBD were immobilized on cellulose and chitin beads, respectively. While the wt enzyme (lacking CBMs) did not immobilize on any beads (data not shown). The two immobilized chimeric proteins showed maximum activity in the range 55–60 °C and pH 4–5 (Figure S3). The obtained cellulose and chitin beads appeared stable under all the conditions used in the enzymatic assays.

**Figure 2:**
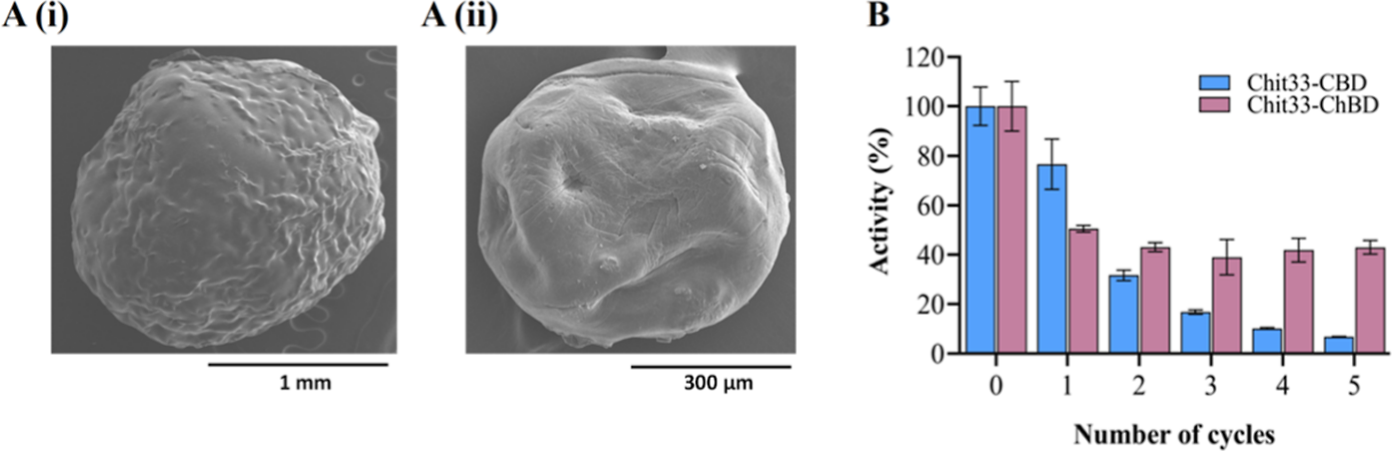
SEM of the generated beads and reusability of the immobilized biocatalysts. (A) SEM images of dry cellulose (i) and chitin (ii) beads; scale bars are shown. (B) Reusability of the referred immobilized chimeras after 30 min cycles. Data are the mean of three individual experiments. Standard errors are indicated. 100% activity refers to 187 and 50 U/g of support for the Chit33-CBD and Chit33-ChBD biocatalysts, respectively.

The reusability of the immobilized biocatalysts was analyzed using batch reactions (Figure 2B). The biocatalyst immobilized on chitin beads retained more than 40% of its initial activity after five cycles of use, while the one immobilized on cellulose beads retained almost 40% of its initial activity only after the second cycle but less than 5% after the fifth.

### 3.4 Activity of Chit33 Variants on Chitinous Substrates and Analysis of Hydrolytic Products

Both the Chit33 wt and its two chimeric variants released reducing sugars from the shrimp (α-allomorph) and squid pen (β-allomorph) of different chitinous materials, including α-/β-CC and various types of α-/β-chitosan differing in size and degree of acetylation. Although to a lesser extent, they also acted on powdered chitin (α-CP). The bar chart presented in Figure 3 shows the relative activity (%) obtained with the three protein variants on the used substrates. Notably, the wt variant showed greater specific activity on β-CC compared to α-CC (3.33 ± 0.04 U/mg versus 2.17 ± 0.02 U/mg; Table S1). The chimera Chit33-CBD showed significantly higher activity than the wt on both CC types and even on the used powder chitin, indicating that the presence of the CBD domain improved the efficiency of the enzyme on these substrates. However, the wt enzyme showed slightly higher activity than its modified variants when using all chitosan types, except with CHIT600, where Chit33-CBD showed the same activity as the wt. Clearly, the fusion of the protein to the ChBD domain reduced the activity of the enzyme on all analyzed substrates.

**Figure 3:**
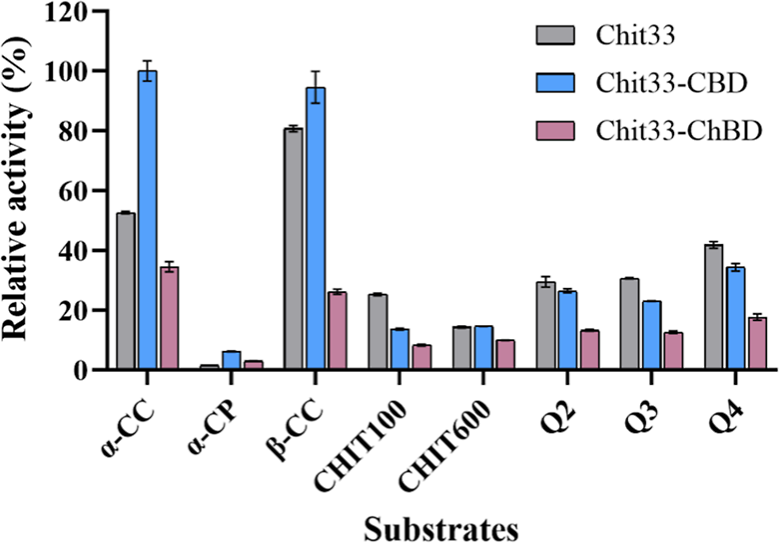
Activity of the Chit33 variants on the indicated chitinous substrates. An activity of 100% corresponds to 4.13 U/mg and was obtained when using the variant Chit33-CBD with α-CC. All substrates were used at 8 mg/mL. α-Chitosan: CHIT100 and CHIT600; β-chitosan: Q2, Q3, and Q4. Data are the average of three independent experiments, and standard errors are indicated.

To further explore the hydrolytic activity of the chimeric enzymes on these substrates, kinetic analyses for the apparent catalytic efficiency were attempted but without success, mainly due to the high viscosity and poor dissolution of the chitinous substrate used. Products formed after one h of hydrolysis were also analyzed using mass spectrometry. In all reactions, masses corresponding to small fully or partially acetylated COS (faCOS and paCOS, respectively) were detected (Table S2). Thus, in most of these reactions mediated by the three chitinase variants containing CC (α/β) and CP (α), faCOS, from 1 to 4 NAG units ((NAG)_1–4_) were predominantly detected, and in some cases, but to a lesser extent paCOS including 1–3 units of both GlcN and NAG ((GlcN)_1–3_-(NAG)_1–3_). Concerning chitosan, mainly (NAG)_1–2_ followed, if detected, by (GlcN)_1–2_-(NAG)_1–3_ were measured from β-chitosans; NAG followed by (GlcN)_1–2_-NAG from α-chitosan CHIT100; and (GlcN)_1–4_-(NAG)_1–2_ together, if detected, with (NAG)_1–4_ from the α-chitosan CHIT600 (Table S2).

### 3.5 Chitin Conversion and Specificity of the Products Formed in 24 h Reactions

Hydrolysis of both α-and β-CC by Chit33, Chit33-CBD, and Chit33-ChBD resulted in a conversion of the initial material into soluble products (COS) of about 27, 35, and 13% (α-CC) and 21, 39, and 9% (β-CC), respectively. In addition, using untreated shrimp shell powder (α-CP) as a substrate resulted in at least a 3-fold reduction in conversion to soluble products for each one of the protein variants (Figure 4A). Thus, the addition of ChBD to Chit33 decreased COS conversion by 22–50% for the three chitin types used in this work, while CBD increased it by 30% for α-CC and 85% for β-CC, with no significant effect on α-CP. Furthermore, by using the wt variant of Chit33, which a priori showed more activity on β-CC than on α-CC (Figure 3), a slightly greater conversion of α-CC was achieved. The composition of amino sugars in the 24 h-reaction mixtures, including this last substrate, was analyzed by MS (Figure 4B and Table S3). As expected, the *endo*-chitinase Chit33 produced basically (NAG)_2–4_ with NAG_4_ being the predominant product for both the wt and the two chimeric variants. In fact, NAG_4_ constituted almost 85% of the COS produced by Chit33-ChBD and about 50% of those produced by both the Chit33-CBD and wt variants. However, while NAG_3_ and NAG_2_ constituted about 35 and 10% of the COS produced by the wt enzyme, they represented only 10 and 12% of those produced by Chit33-CBD, respectively. Notably, only Chit33-CBD produced significant amounts of (NAG)_6–9_, whereas only traces of COS greater than NAG_4_ were detected when using Chit33 and Chit33-ChBD. Additionally, MS analysis also detected masses corresponding to partially acetylated COS in the reaction products (Table S3). Thus, pointing to the addition of the binding domains to Chit33 influences the diversity of the substrate hydrolysis sites and, therefore, that of the products formed. In this way, and while the addition of the ChBD restricted the production of total COS, predominantly yielding NAG_4_ at the expense of NAG_3_, the incorporation of CBD maintains the production of NAG_4_, reduces that of NAG_3_, and broadens the product profile, apparently resulting in higher quantities of longer COS (NAG)_5–9_.

**Figure 4:**
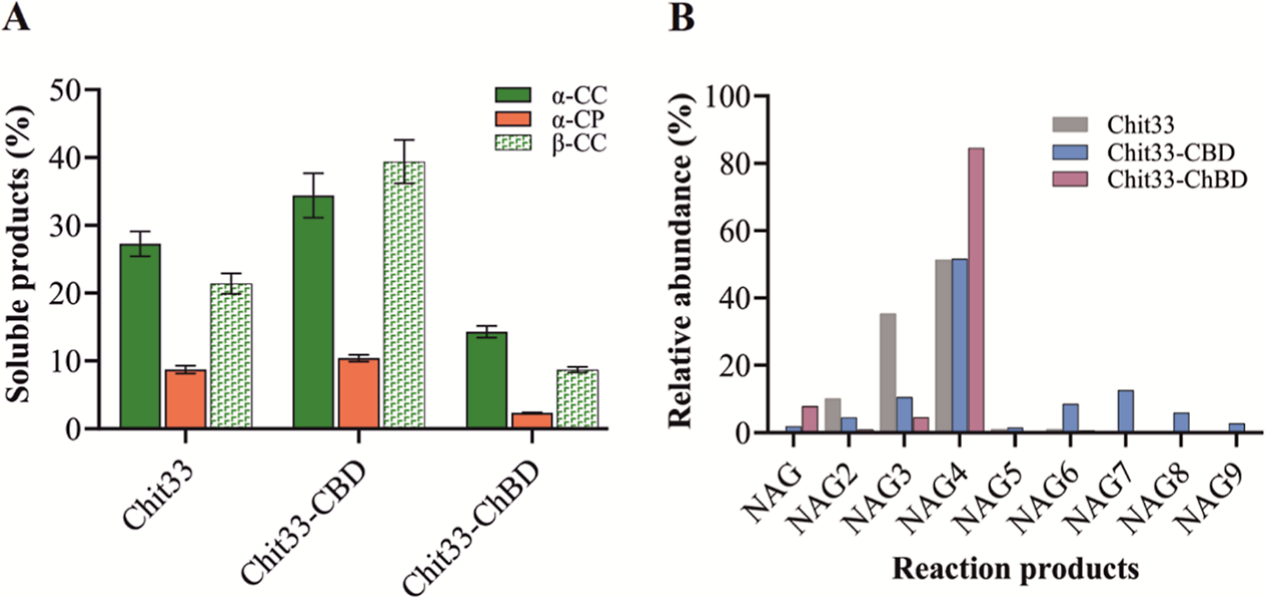
Conversion of chitin into soluble products by the 3 chitinase variants and faCOS profile formed from α-CC. (A) Soluble products (%) were calculated based on the mass balance of the nonsolubilized materials; 7.9 μg of the three-protein variant were used in this assay. (B) Relative abundance of the indicated faCOS referred to total NAG derivatives detected by MS. Only peaks corresponding to COS with a DP of 1–9 units of NAG were represented.

## 4 Discussion

Different chitinases of GH18 fused to CBMs, typically at its C-terminal ends, have already been expressed in heterologous systems. However, most of those fused at the N-terminal ends reported no activity.^19,32,33^ In this context, one of the best studied examples was the *exo*-chitinase Chit46 from T. harzianum fused to CBM family 3 (CBM3), which was expressed in P. pastoris to about 36 U/mL with the excellent specific activity of ∼76 U/mg (31.4 U/mL and 34.5 U/mg) for the wt variant.^32^ However, as far as we know, only a few *endo*-chitinases fused to these types of modules have been obtained and analyzed in detail. Furthermore, the already reported chimeric chitinases always demanded a laborious purification step prior to their use.^10,11,16,18^ For example, the referred Chit46 required chromatographic purification with two columns (HisTrap HP and Sephadex G-75),^32^ whereas in our work, despite the low protein production level obtained, this step was apparently reduced to a simple concentration of the yeast extracellular medium (Figure S6).

The two Chit33 chimeric proteins obtained in this work showed a higher optimal temperature range than the wt variant. Changes in optimal temperatures have already been detected with the fusion of CBMs to different chitinases. For example, the addition of CBM family 6 (CBM6) to the commented Chit46 increased its optimal temperature range from 40 to 50 °C to 37–58 °C, and CBM family 92 (CBM92) to Chit18 from Chitinophaga pinensis retained >50% of this protein activity at 70 °C.^32,34^ In addition, the chimeric *endo*-cellulase CelE-CBM family 64 (CBM64) also showed higher cellulolytic activity than its wt variant,^15^ and more recently, fusion of CBMb1 to the pullulanase from Deinococcus radiodurans increased its activity on raw waxy corn starch by 60%.^35^

Immobilizing enzymes enhances their stability against temperature and pH fluctuations, making them ideal for industrial green bioprocesses and sustainable biotechnology applications. Thus, a wide variety of enzymes, lipases, lactases, invertases, among others, and some chitosanases and chitin deacetylases have already been immobilized on cellulose and chitin beads using CBMs, with 30–80% of the initial activity retained.^36–39^ However, to our knowledge, no chitinase had yet been immobilized to them. The immobilization of above enzymes barely altered its biochemical properties compared to the free enzymes, and the slight variations detected on their optimal temperatures and pHs were associated with possible conformational changes of the proteins after their immobilization.^21,40–42^

The biocatalysts immobilized on the two support types used in this work showed a loss of activity after successive cycles of use. This phenomenon has already been widely discussed in the literature, and factors such as conformational changes, substrate inhibition, leaching, and physical wear, among others, may contribute to it.^43,44^ However, the three Chit33 protein variants used here apparently bound to both cellulose and chitin beads. Thus, the apparent dissociation constants (*K*_d_) of wt Chit33 to cellulose and chitin beads were 12.0 ± 3.4 and 126.1 ± 31.5 μM, respectively, and addition of both CBD and ChBD significantly decreases these values. Chit33-CBD and Chit33-ChBD showed *K*_d_ values of 5.2 ± 2.1 and 5.2 ± 1.8 μM on cellulose beads and 20.5 ± 8.2 and 20.6 ± 6.5 μM on chitin beads, respectively (Figure S7). The same binding affinity increment effect was observed when the GH19 chitinase ChiC from Streptomyces griseus was fused with a ChBD.^45^

To further investigate the structural basis of this effect, the putative folding of the two chimeric constructs, including CBD and ChBD, was analyzed by using the AI-based structure prediction program AlphaFold2. Figure 5 shows the best models generated, both exhibiting a high overall quality, as evaluated by the server (mean predicted local distance difference, pLDDT, 85.5 and 87.7 for variants including CBD and ChBD, respectively). However, the long segment linking the two domains was predicted to be a highly disordered region, as seen in the assigned low pLDDT values (Figure 5A,D), indicating that their exact position is variable. This is reflected in the corresponding Predicted Aligned Error (PAE) plots (Figure 5C,F), which gives some uncertainty in the relative orientation of the two domains within each construct. Therefore, a high internal flexibility of the two variants is assumed, which makes it difficult to have a clear picture of the precise conformation that binds the substrate in each case. Nevertheless, all the generated models suggest that the two additional domains approach the catalytic domain facing their binding site to the Chit33 active site (Figure 5B,E), i.e., exposing the aromatic residues conserved within families CBM1 (Chit33-CBD) and CBM18 (Chit33-ChBD) that are responsible for binding the substrate. In CBMs-type A, including CBM1, these aromatic residues form a flat, hydrophobic surface that facilitates stacking interactions with the sugar rings in the substrate, contributing to effective binding to crystalline surfaces. In this context, residues W13, Y39, and Y40 of CBD would be well positioned to contribute to orienting the substrate toward the active site (Figure 5B). Moreover, the higher hydrolytic activity on chitin detected in the Chit33-CBD variant (Table 1) possibly suggests that the CBD domain was able to accommodate the substrate in a more favorable orientation to interact with the catalytic site than the ChBD domain (Figure 5E). Conversely, the CBD domain is possibly less accessible to the cellulose support compared with ChBD with chitin, explaining the superior capability of fixing Chit33-ChBD to the chitin beads (Figure 2B).

**Figure 5:**
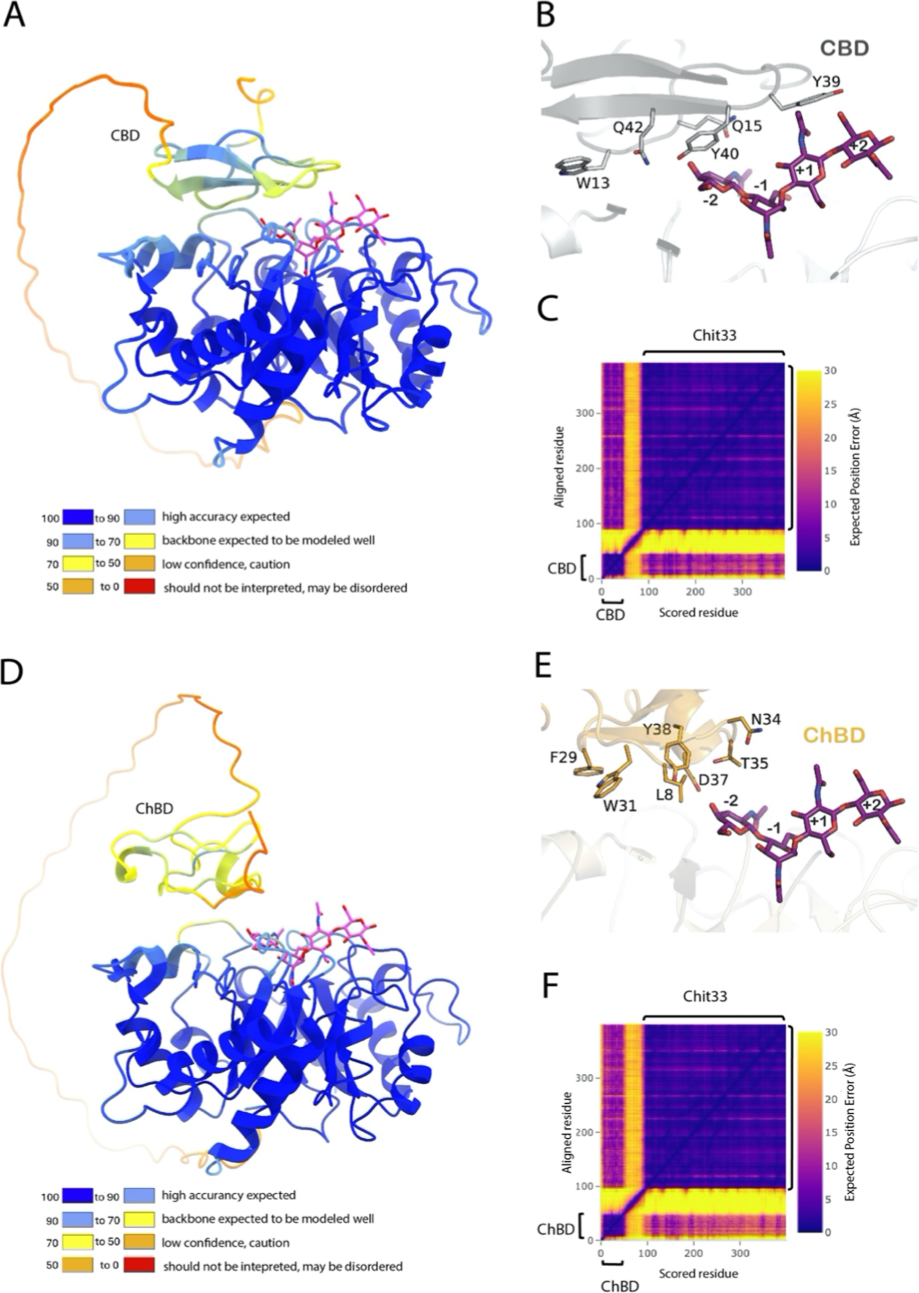
Models of the Chit33-CBD (A) and Chit33-ChBD (D) variants, colored by local confidence, estimated with the predicted local distance test (pLDDT), as returned by Alphafold2. A NAG4 substrate bound to the reported Chit33-NAG4 complex (PDB code7ZY9) is shown as sticks. (B,E) Zoom of the models showing as sticks the residues of the additional domains that might contribute to substrate binding. (C,F) Confidence of interdomain orientation estimated with the PAE in Å between all pairs of residues in the protein, as returned by Alphafold2.

Altogether, this indicates that although the two CBMs fused to Chit33 allow the chimeric proteins to bind to the generated supports, ChBD not only provides a more stable immobilization than CBD but also reduces its activity, which cannot be explained structurally given the predicted flexibility generated by the highly disordered linker organization. This stability a priori could also make Chit33-ChBD more cost-effective by enabling reuse up to five times, partially offsetting its lower activity.

Although the Chit33 variants showed activity on chitin deacetylated forms, the two chimeric proteins hydrolyzed chitosan with less efficiency in comparison with the wt. This decrease in the activity of GH18 chitinases after their fusion to CBMs had already been previously reported, highlighting both the implication of the distance between these modules and the enzyme catalytic domain and the effect of their positioning at either the protein C- or N-terminal. Thus, all the variants of the C-terminal CBM-fused Chit46 from T. harzianum showed chitinase activity, but none of the N-terminal-fused proteins exhibited it,^32^ suggesting that fusion to CBMs could also difficult someway the access to the catalytic site of the chimeric protein.

Overall, our results indicate that the CBD domain significantly enhances the activity of Chit33 on different substrate types, particularly the colloidal and crystalline forms of chitin. Unlike ChBD (a CBM-type C), CBD (a CBM-type A) recognizes crystalline surfaces of insoluble polysaccharides (substrates) and typically possesses flat binding faces. As already reported, α-CC possesses higher crystallinity than β-CC, which may also contribute to explain the apparent preference of Chit33-CBD for α-CC.^46,47^ On the other hand, the Chit33-CBD variant appears less affected by substrate conformation than the other two variants, and therefore, we cannot rule out the possibility that the CBD may guide Chit33 toward the chitin substrate, although it probably binds less tightly to it than ChBD. This could result in a “binding and unbinding” cycle that provides Chit33-CBD access to additional cleavage sites along the chains. Although CBMs are known to recognize polysaccharides and facilitate degradation by keeping the biocatalyst near the substrate for a longer time, substrate choice significantly affects the activity of the protein variants. A similar effect was observed with the chitinases ActChi and Chit46 from the Actinomycetes bacterium and T. harzianum, respectively, as well as the cellobiohydrolase Ce17A from T. harzianum, after their fusion to CBMs.^32,33,48^ In addition, no activity improvement was observed when using the deacetylated form of chitin, which might be due to its lower crystallinity, resulting from a higher degree of deacetylation and altered acetylation patterns (making chitosan more soluble in acidic conditions). Furthermore, since there is no enhanced binding of the chimeric enzymes to chitosan, their hydrolytic efficiency on these substrates was reduced. In this context, chitinase ChiJ from Bacillus sp. also lost 60% of its binding capacity when chitosan was used instead of chitin.^49^ The effect of the substrate deacetylation on the chitinase activity has been previously studied, and the activity of different chitinases GH18 reported to be strongly dependent on the acetylated glucosamine residue positioned in the −1 catalytic subsite during the breakdown of glycosidic bond. Thus, deacetylation reduces the efficiency of chitinase by altering the substrate structure and the enzyme binding capacity, leading to decreased enzyme activity on chitosan compared to chitin.^22,50^ Concerning the hydrolytic preference of the three Chit33 variants toward acidic pretreated α-chitin (CC) over CP, these results have already been reported by using the chitinase from Stenotrophomonas rhizophila G22 and that from Paenibacillus chitinolyticus (*exo*-chitinase CHI), which showed 80 and 50% more activity on CC than on CP, respectively.^51,52^

Our results suggest that the addition of the CBM to Chit33 clearly influences the diversity of the substrate hydrolysis sites and therefore the products formed. Although the influence of CBMs on product profiles is not well documented, that of the *endo*-mannanase Man5A (GH5) from Clostridium thermocellun was clearly affected by the addition of CBM family 32 (CBM32; a mannan binding module), differing the products obtained from mannotetraose and mannopentaose.^53^ In addition, and more recently, the CBM6 of two bacterial GH5_34 subfamily arabinoxylanases was also swapped, altering their product profile. The two hybrid proteins exhibit distinct activity on hemicellulose arabinoxylan, yielding different reaction product patterns, despite their high sequence identity, conserved active sites, and similar domain composition.^54^

The integration of CBMs into chitinase constructs significantly enhances their catalytic properties, particularly by improving substrate binding and fine-tuning specificity. Considering altogether these results, which demonstrate that variant including the CBD broadened product diversity and achieved higher conversion rates on both α- and β-chitin, while that including ChBD predominantly yielded NAG_4_ with only traces of NAG_1–3_, the CBM choice seems crucial. Furthermore, since specific polymerization and acetylation levels influence the biological properties of the formed products, further engineering of these carbohydrate binding domains together with the substrate selection could enhance the efficiency and specificity of the catalyzed chitinase-based processes, a goal of interest for the health, food, and agricultural industry.

## Supplementary Material



## Notes

Experimental conditions to produce chitosan from squid pen byproducts; scheme showing the processes of immobilization and reuse of the biocatalysts; effect of temperature and pH of the generated Chit33 variants soluble or immobilized; Michaelis–Menten kinetic analyses of the variants on α-colloidal chitin; chitin and cellulose beads on a millimeter background; specific activity of the referred Chit33 variants; main peaks and intensities of the MS corresponding to the reaction mixtures after 1 h; main peaks and intensities of the MS when using Chit33 in the presence and absence of CBMs on alpha CC after 24 h; SDS-PAGE analysis of the concentrated Chit33 variants; and dissociation constants (*K*_d_) fits for the binding of Chit33 protein to cellulose and chitin beads (PDF)

## Abbreviations

GH: glycosyl hydrolase
CC: colloidal chitin
CP: chitin powder
COS: chitooligosaccharides
paCOS: partially acetylated chitooligosaccharides
faCOS: fully acetylated chitooligosaccharides
NAG: *N*-acetyl-d-glucosamine
GlcN: d-glucosamine
BB: binding buffer
CBD: cellulose binding domain
ChBD: chitin binding domain
CBM: carbohydrate binding module
wt: wild-type
