# Supplementary material for: Boosting Biocatalytic Efficiency: Engineering of Chitinase Chit33 with Chitin and Cellulose Binding Domains for Sustainable Chitin Conversion
Source: J Agric Food Chem. 2025 Apr 25;73(18):11121–31. doi: 10.1021/acs.jafc.4c10364 (PMC12129251; doi:10.1021/acs.jafc.4c10364)
Supplement: Supplementary file 1 [file jf4c10364_si_001.pdf]

Supporting information

Boosting Biocatalytic Efficiency: Engineering of chitinase Chit33 with Chitin and Cellulose Binding Domains for Sustainable Chitin Conversion

María Martínez-Ranz[a], Peter E Kidibule[a,b,]*, Elena Jiménez-Ortega[c], Jesús Valcárcel[d], José Antonio Vázquez[d], Julia Sanz-Aparicio[c], María Fernández-Lobato[a,]*

[a] *Department of Molecular Biology, Centre of Molecular Biology Severo Ochoa, CSIC-UAM, 28049 Madrid, Spain*

[b] *Department of Chemistry Biotechnology and Food Science, Norwegian University of Life Sciences, P.O. Box 5003, N-1432 Ås, Norway*

[c] *Department of Crystallography and Structural Biology, Institute of Physical Chemistry Blas Cabrera, CSIC, 28006 Madrid, Spain*

[d] *Recycling and Valorisation of Waste Materials Group (REVAL), Institute of Marine Research, IIM-CSIC, 36208 Galicia, Spain*

**Figure S1**	Flowchart summarizing the experimental conditions to produce chitosan from squid pen by-products.
**Figure S2**	Scheme showing the processes of immobilization and reuse of the biocatalysts.
**Figure S3**	The effect of temperature and pH of the generated Chit33 variants activity. Soluble or immobilized biocatalysts.
**Figure S4**	Michaelis-Menten kinetic analyses of the Chit33 variants on α-colloidal chitin.
**Figure S5**	Image of the chitin and cellulose beads on millimeter background.
**Table S1**	Specific activity of the referred Chit33 variants on the indicated substrates.
**Table S2**	Main peaks and intensities of the MS corresponding to the reaction mixtures obtained with the indicated substrates and protein variants after 1 h.
**Table S3**	Identified peaks and intensities of the mass spectrum when using Chit33 in the presence and absence of CBMs on colloidal alpha chitin.
**Figure S6**	SDS-PAGE analysis of the concentrated Chit33 variants previously expressed in *P. pastoris*.
**Figure S7**	Dissociation constants (K_d) fits for the binding of Chit33 protein variants to cellulose and chitin beads.

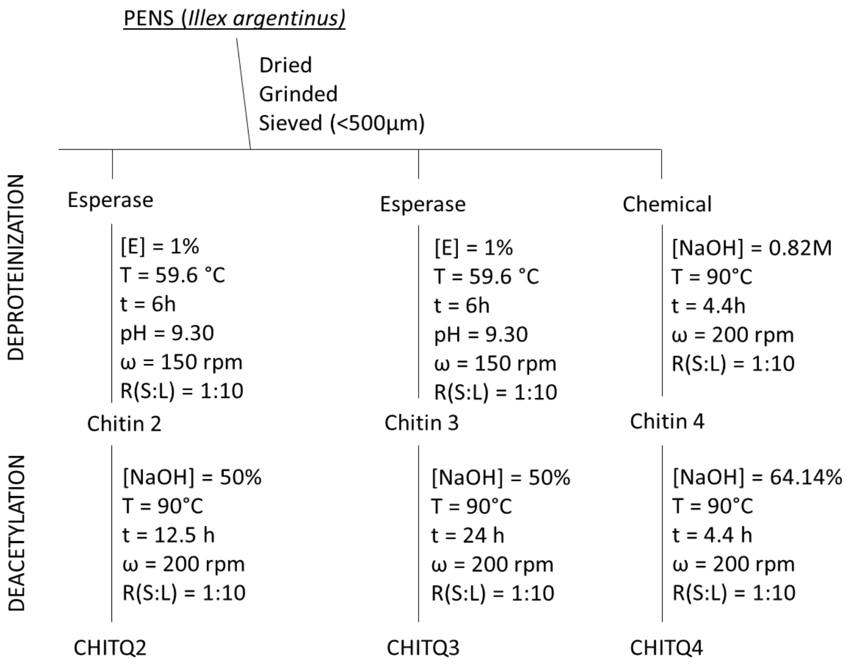

Figure S1. Flowchart summarizing the experimental conditions to produce chitosan from squid pen by-products. The combination of chemical and enzymatic treatments used is indicated.

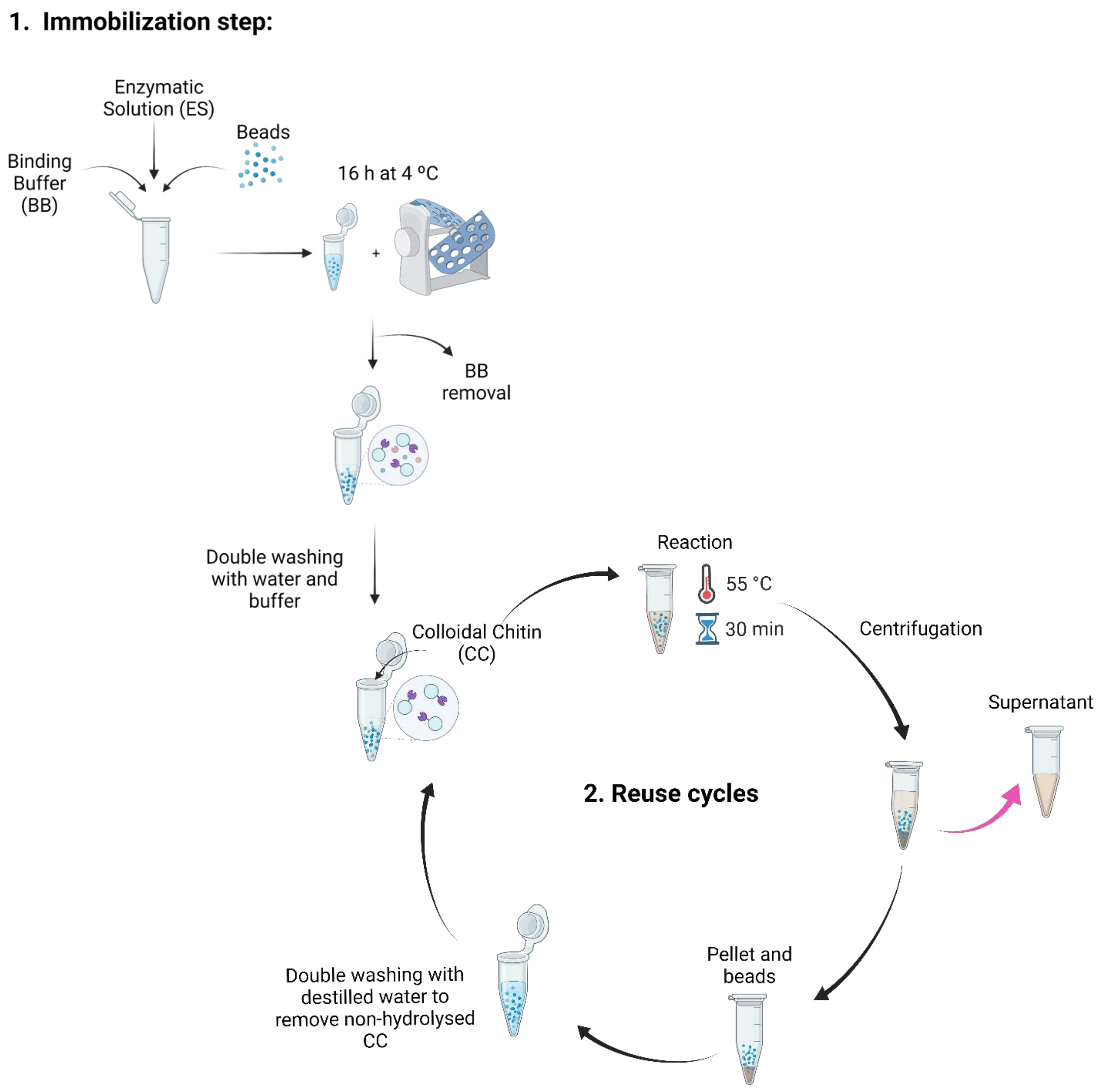

Figure S2. Scheme showing the processes of immobilization and reuse of the biocatalysts. The reaction conditions were set at an intermediate temperature between the optimum of the two enzyme variants.

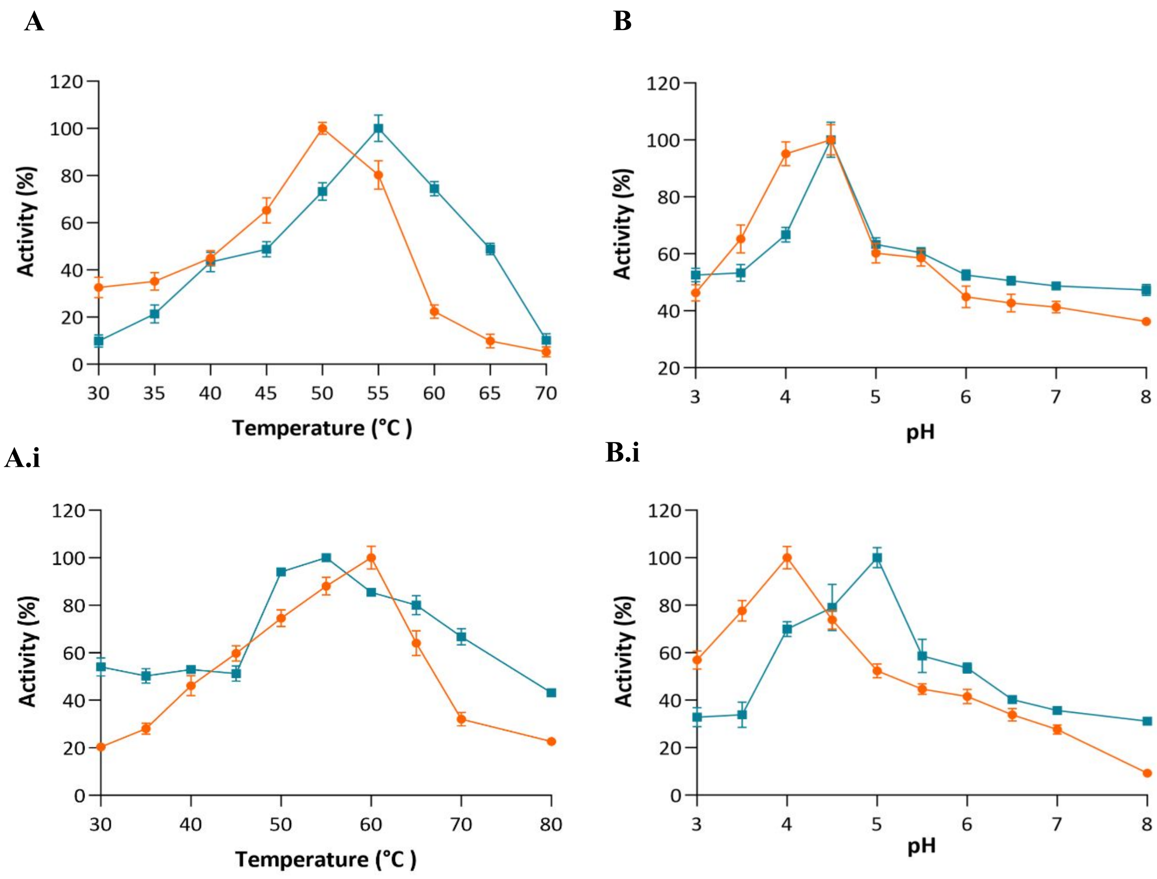

Figure S3. The effect of temperature and pH on the generated Chit33 variants activity. Soluble or immobilized biocatalysts. Chit33-CBD (orange) and Chit33-ChBD (blue) chitinolytic activity was evaluated in its soluble (top panels; A and B) and immobilized form (lower panels; A.i and B.i) using colloidal chitin as substrate. The optimum temperature was evaluated at pH 5 and the optimum pH at 45 ºC for free and immobilized enzymes since they were the optimal conditions for the wt variant. Assays were conducted in triplicate and data are means of three parallel measurements. Standard errors are indicated. 100% activity refers in soluble variants CBD and ChBD to 700-122 mU/mL for temperature, and 467.87-93.64 mU/mL for pH. As for their immobilized form, 187 and 50 U/g of support for Chit33-CBD and Chit33-ChBD, respectively.

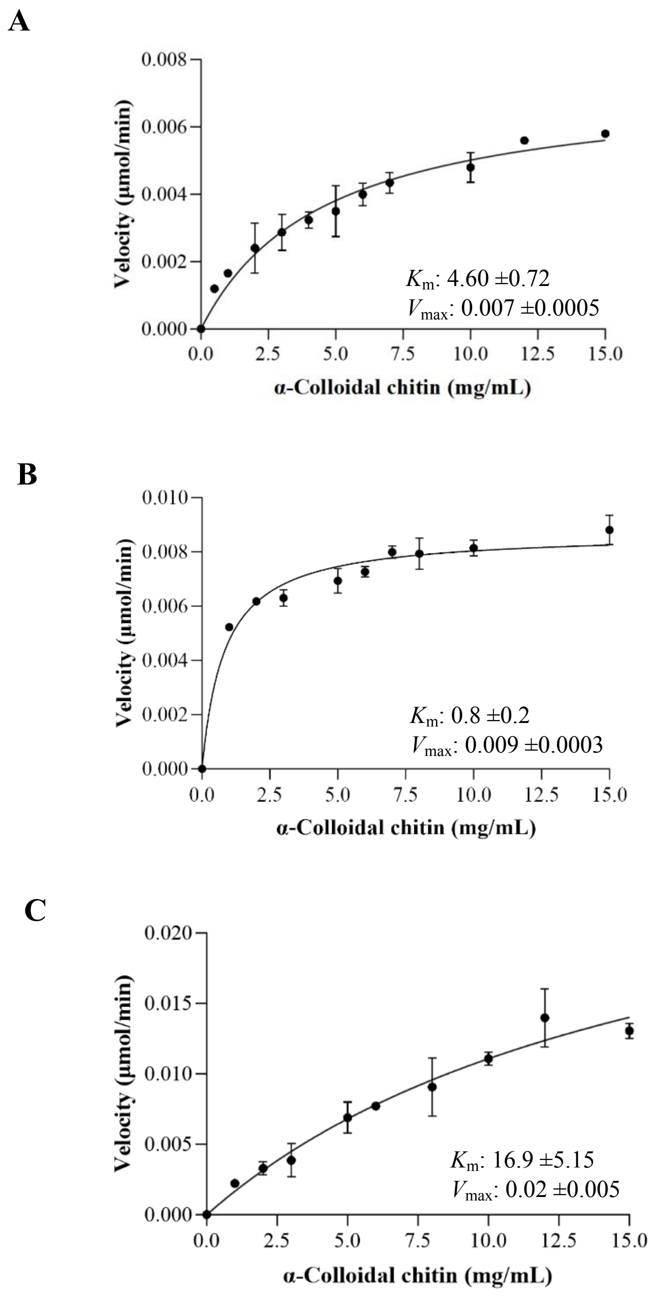

Figure S4. Michaelis-Menten kinetic analyses of the Chit33 variants on α-colloidal chitin. Chit33 wild-type: 1.97x10^{-5} µmol (A), Chit33-CBD: 9.08x10^{-5} µmol (B) and Chit33-ChBD: 9.85x10^{-5} µmol (C) were used this assay. Apparent *K*m and Vmax values are expressed in mg/mL and µmol/min (in a 1 mL reaction), respectively.

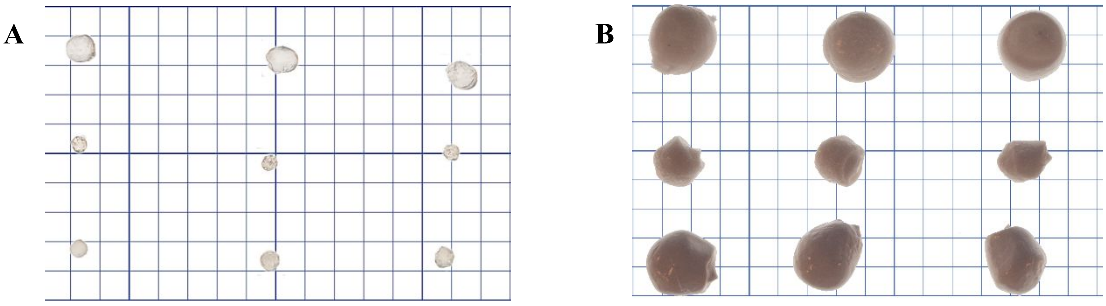

Figure S5. Image of the chitin and cellulose beads on millimeter background. For the chitin (A) and cellulose (B) beads, and starting from the top, those in the first row were triplicates preserved in water, those in the second lyophilized and those in the third dried at 37 °C during 16 h.

Table S1. Specific activity of the referred Chit33 variants on the indicated substrates

	**Chit33**	**Chit33-CBD**	**Chit33-ChBD**
**α- CC**	2.17 ±0.02	4.13 ±0.1	1.42 ±0.02
**α- CP**	0.06 ±0.003	0.26 ±0.01	0.12 ±0.001
**β- CC**	3.33 ±0.04	3.90 ±0.22	1.08 ±0.03
**CHIT100**	1.05 ±0.02	0.56 ±0.01	0.34 ±0.02
**CHIT600**	0.59 ±0.01	0.06 ±0.0003	0.41 ±0.005
**Q2**	1.22 ±0.07	1.09 ±0.03	0.55 ±0.02
**Q3**	1.27 ±0.01	0.95 ±0.005	0.52 ±0.02
**Q4**	1.73 ±0.05	1.42 ±0.05	0.73 ±0.04

Specific activity in U/mg is indicated. Data are the average of 3 independent measures and standard errors are indicated.

Table S2. Main peaks and intensities of the mass spectrum corresponding to the reaction mixture obtained with different substrates and protein variants after 1h.

Chit33 wt				
	**m/z**	**Intensity (counts)**	**% Intensity**	**Identified COS**
**α-CC**	223.984	1076	6.09	[NAG + 2H]+
	405.334	2403	13.60	[GlcN-NAG + Na]+
	425.133	2849	16.13	[NAG2 + H]+
	447.335	1573	8.90	[NAG2 + Na]+
	566.399	2582	14.62	[GlcN2-NAG + Na]+
	608.415	1455	8.24	[GlcN-NAG2 + Na]+
	727.496	2104	11.91	[GlcN3-NAG + Na]+
	769.512	537	3.04	[GlcN2-NAG2 + Na]+
	888.609	1460	8.26	[GlcN4-NAG + Na]+
	1049.732	903	5.11	[GlcN5-NAG + Na]+
	1210.853	484	2.74	[GlcN6-NAG + Na]+
	1371.974	240	1.36	[GlcN7-NAG + Na]+
**α-CP**	224.029	536	5.45	[NAG + 3H]+
	425.224	1671	17.00	[NAG2 + H]+
	447.426	3558	36.21	[NAG2 + Na]+
	608.525	811	8.25	[GlcN-NAG2 + Na]+
	650.552	2362	24.04	[NAG3 + Na]+
	853.716	889	9.05	[NAG4 + Na]+
**β-CC**	223.981	822	13.49	[NAG + 2H]+
	244.32	582	9.55	[NAG + Na]+
	425.114	257	4.22	[NAG2 + H]+
	447.304	1393	22.87	[NAG2 + Na]+
	650.406	1416	23.24	[NAG3 + Na]+
	853.554	1622	26.63	[NAG4 + Na]+
**CHIT600**	405.474	1253	10.76	[GlcN-NAG + Na]+
	425.297	976	8.38	[NAG2 + H]+
	566.574	2575	22.10	[GlcN2-NAG + Na]+
	608.581	1020	8.76	[GlcN-NAG2 + Na]+
	727.687	2308	19.81	[GlcN3-NAG + Na]+
	769.701	642	5.51	[GlcN2-NAG2 + Na]+
	831.528	798	6.85	[NAG4 + H]+
	888.820	1426	12.24	[GlcN4-NAG + Na]+
	1049.958	652	5.60	[GlcN5-NAG + Na]+
**CHIT100**	224.302	4891	53.22	[NAG + 3H]+
	405.454	2255	24.54	[GlcN-NAG + Na]+
	566.506	1190	12.95	[GlcN2-NAG + Na]+
	727.597	555	6.04	[GlcN3-NAG + Na]+
	888.712	209	2.27	[GlcN4-NAG + Na]+
	1049.75700	90	0.98	[GlcN5-NAG + Na]+
**Q2**	223.924	2151	71.32	[NAG + 2H]+
	385.028	139	4.61	[GlcN-NAG + 3H]+
	447.238	407	13.49	[NAG2 + Na]+
	608.323	190	6.30	[GlcN-NAG2 + Na]+

	650.335	129	4.28	[NAG3 + Na]+
**Q3**	267.416	1238	100	[NAG + 2Na]+
**Q4**	223.961	2632	77.64	[NAG + 2H]+
	447.232	570	16.81	[NAG2 + Na]+
	650.308	188	5.55	[NAG3 + Na]+
	811.404	98.2	2.90	[GlcN-NAG3 + Na]+

**Chit33-CBD**				
	**m/z**	**Intensity (counts)**	**% Intensity**	**Identified COS**
**α-CC**	244.396	3114	8.86	[NAG + Na]+
	425.274	291	0.83	[NAG2 + H]+
	447.485	10533	29.97	[NAG2 + Na]+
	608.623	248	0.71	[GlcN-NAG2 + Na]+
	650.654	5251	14.94	[NAG3 + Na]+
	811.820	388	1.10	[GlcN-NAG3 + Na]+
	853.878	15319	43.59	[NAG4 + Na]+
**α-CP**	447.439	4512	24.82	[NAG2 + Na]+
	608.562	865	4.76	[GlcN-NAG2 + Na]+
	650.591	3869	21.29	[NAG3 + Na]+
	811.748	659	3.63	[GlcN-NAG3 + H]+
	853.783	8272	45.51	[NAG4 + Na]+
**β-CC**	224.029	6341	67.62	[NAG + 3H]+
	447.292	1563	16.67	[NAG2 + Na]+
	650.384	1241	13.23	[NAG3 + Na]+
	853.529	232	2.47	[NAG4 + Na]+
**CHIT600**	224.371	3386	19.97	[NAG + 3H]+
	405.517	3145	18.55	[GlcN-NAG + Na]+
	566.505	3373	19.89	[GlcN2-NAG + Na]+
	727.599	3298	19.45	[GlcN3-NAG + Na]+
	888.731	2362	13.93	[GlcN4-NAG + Na]+
	1049.869	1392	8.21	[GlcN5-NAG + Na]+
**CHIT100**	224.077	6113	79.36	[NAG + 3H]+
	405.315	411	5.34	[GlcN-NAG + Na]+
	447.322	293	3.80	[NAG2 + Na]+
	566.391	276	3.58	[GlcN2-NAG + Na]+
	608.410	158	2.05	[GlcN-NAG2 + Na]+
	727.500	163	2.12	[GlcN3-NAG + Na]+
	815.219	289	3.75	[GlcN2-NAG2 + 3Na]+
	888.626	96.3	1.25	[GlcN4-NAG + Na]+
**Q2**	223.985	8292	76.51	[NAG + 2H]+
	385.13	287	2.65	[GlcN-NAG + 3H]+
	447.255	1792	16.53	[NAG2 + Na]+
	608.332	274	2.53	[GlcN-NAG2 + Na]+
	650.342	193	1.78	[NAG3 + Na]+
	811.442	92.4	0.85	[GlcN-NAG3 + H]+

Q3	224.332	38410	81.56	[NAG + 3H]+
	425.552	3383	7.18	[NAG2 + H]+
	470.292	2675	5.68	[NAG2 + 2Na]+
	815.234	2202	4.68	[GlcN2-NAG2 + 3Na]+
	831.215	423	0.90	[NAG4 + H]+
Q4	223.96	3291	52.66	[NAG + 2H]+
	385.094	173	2.77	[GlcN-NAG + 3H]+
	447.238	1813	29.01	[NAG2 + Na]+
	608.307	498	7.97	[GlcN-NAG2 + Na]+
	650.323	347	5.55	[NAG3 + Na]+
	811.415	128	2.05	[GlcN-NAG3 + H]+

**Chit33-ChBD**				
	**m/z**	**Intensity (counts)**	**% Intensity**	**Identified COS**
**α-CC**	244.357	2114	16.67	[NAG + Na]+
	425.321	469	3.70	[NAG2 + H]+
	447.427	3361	26.50	[NAG2 + Na]+
	650.582	1375	10.84	[NAG3 + Na]+
	811.829	151	1.19	[GlcN-NAG3 + Na]+
	853.768	2629	20.73	[NAG4 + Na]+
	244.357	2114	16.67	[NAG + Na]+
	425.321	469	16.67	[NAG2 + H]+
**α-CP**	223.988	847	18.96	[NAG + 2H]+
	425.255	849	19.00	[NAG2 + H]+
	447.425	893	19.99	[NAG2 + Na]+
	608.531	270	6.04	[GlcN-NAG2 + Na]+
	853.761	1609	36.01	[NAG4 + Na]+
**CHIT600**	223.984	1076	6.09	[NAG + 2H]+
	405.334	2402	13.60	[GlcN-NAG + Na]+
	425.133	2849	16.13	[NAG2 + H]+
	447.335	1573	8.90	[NAG2 + Na]+
	566.399	2582	14.62	[GlcN2-NAG + Na]+
	608.415	1455	8.24	[GlcN-NAG2 + Na]+
	727.496	2104	11.91	[GlcN3-NAG + Na]+
	769.512	537	3.04	[GlcN2-NAG2 + Na]+
	888.609	1460	8.26	[GlcN4-NAG + Na]+
	1049.73	903	5.11	[GlcN5-NAG + Na]+
	1210.85	484	2.74	[GlcN6-NAG + Na]+
	1371.97	240	1.36	[GlcN7-NAG + Na]+
**CHIT100**	224.04	4313	61.30	[NAG + 3H]+
	405.318	626	8.90	[GlcN-NAG + Na]+
	447.324	373	5.30	[NAG2 + Na]+
	566.392	565	8.03	[GlcN2-NAG + Na]+
	608.411	313	4.45	[GlcN-NAG2 + Na]+
	727.503	382	5.43	[GlcN3-NAG + Na]+
	769.526	159	2.26	[GlcN2-NAG2 + Na]+
	888.626	202	2.87	[GlcN4-NAG + Na]+

	1049.76	103	1.46	[GlcN5-NAG + Na]+
Q2	223.985	8292	76.51	[NAG + 2H]+
	385.13	287	2.65	[GlcN-NAG + 3H]+
	447.255	1792	16.53	[NAG2 + Na]+
	608.332	274	2.53	[GlcN-NAG2 + Na]+
	650.342	193	1.78	[NAG3 + Na]+
	811.442	92.4	0.85	[GlcN-NAG3 + H]+
Q3	224.045	16560	94.10	[NAG + 3H]+
	447.239	1039	5.90	[NAG2 + Na]+
Q4	224.025	17023	68.72	[NAG + 3H]+
	385.173	513	2.07	[GlcN-NAG + 3H]+
	405.245	478	1.93	[GlcN-NAG + 2Na]+
	447.251	5532	22.33	[NAG2 + Na]+
	608.3	777	3.14	[GlcN-NAG2 + Na]+
	769.373	147	0.59	[GlcN2-NAG2 + Na]+
	811.393	176	0.71	[GlcN-NAG3 + Na]+
	815.052	126	0.51	[GlcN2-NAG2 + 2Na]+

Data obtained in optimal pH and temperature conditions for the indicated Chit33 variants. Only main $[M+H]^+$ and $[M+Na]^+$ peaks, in the referred mass interval, detected in positive mode are indicated. Relative percentage based on “Intensity (counts)”.

Table S3. Main peaks and intensities of the mass spectrum corresponding to the reaction mixture including α-colloidal chitin after 24 h.

Chit33 wt

m/z	Intensity (counts)	% Intensity	Identified COS
222.084	2066.0	1.91	[NAG + H]+
244.079	560.0	0.52	[NAG + Na]+
425.232	1968.0	1.82	[NAG2 + H]+
447.207	10907.5	10.09	[NAG2 + Na]+
586.328	536.0	0.50	[GlcN-NAG2 + H]+
608.289	675.0	0.62	[GlcN-NAG2 + Na]+
628.338	4807.0	4.44	[NAG3 + H]+
650.311	32150.0	29.73	[NAG3 + Na]+
789.401	1055.0	0.98	[GlcN-NAG3 + H]+
811.372	2801.5	2.59	[GlcN-NAG3 + Na]+
831.41	2720.0	2.52	[NAG4 + H]+
853.388	42317.0	39.13	[NAG4 + Na]+
992.46	136.0	0.13	[GlcN-NAG4 + H]+
1014.425	448.0	0.41	[GlcN-NAG4 + Na]+
1056.451	885.8	0.82	[NAG5 + Na]+
1153.573	108.0	0.10	[GlcN2-NAG4 + H]+
1091.545	134.75	0.12	[GlcN4-NAG2 + Na]+
1175.51	122.0	0.11	[GlcN2-NAG4 + Na]+
1195.538	173.0	0.16	[GlcN-NAG5 + H]+
1210.591	107.0	0.10	[GlcN6-NAG + Na]+
1217.502	309.0	0.29	[GlcN-NAG5 + Na]+

1259.499	923.0	0.85	[NAG6 + Na]+
1378.627	140.8	0.13	[GlcN-NAG5 + Na]+
1398.58	113.8	0.11	[GlcN-NAG6 + H]+
1420.547	282.0	0.26	[GlcN-NAG6 + Na]+
1462.55	604.0	0.56	[NAG7 + Na]+
1520.756	355.0	0.33	[GlcN4-NAG4 +2Na]+
1581.687	158.0	0.15	[GlcN2-NAG6 + Na]+
1623.637	158.0	0.15	[GlcN-NAG7 + Na]+
1665.606	231.0	0.21	[NAG8 + Na]+
1881.826	135.0	0.12	[GlcN4-NAG6 + H]+
2044.07	60.0	0.06	[GlcN5-NAG6 + 3H]+

Chit33-CBD

m/z	Intensity (counts)	% Intensity	Identified COS
222.093	1525.0	1.44	[NAG + H]+
425.254	741.0	0.70	[NAG2 + H]+
447.216	2392.0	2.26	[NAG2 + Na]+
608.419	945.5	0.89	[GlcN-NAG2 + Na]+
628.353	1116.5	1.06	[NAG3 + H]+
650.314	5233.0	4.95	[NAG3 + Na]+
705.424	848.5	0.80	[GlcN3-NAG + H]+
789.405	1609.5	1.52	[GlcN-NAG3 + H]+
831.435	2072.0	1.96	[NAG4 + H]+
853.393	28294.8	26.78	[NAG4 + Na]+
992.554	1796.0	1.70	[GlcN-NAG4 + H]+
1014.531	1531.0	1.45	[GlcN-NAG4 + Na]+
1055.531	619	0.59	[NAG5 + Na]+
1070.58	1600.0	1.51	[GlcN4-NAG2 + 2H]+
1133.615	3388.0	3.21	[GlcN3-NAG3 + Na]+
1188.642	4047.5	3.83	[GlcN6-NAG + H]+
1210.624	1754.0	1.66	[GlcN6-NAG + Na]+
1252.592	3693.5	3.50	[GlcN5-NAG2 + Na]+
1258.590	2995.5	2.84	[NAG6 + Na]+
1272.606	1084.5	1.03	[GlcN4-NAG3 + H]+
1314.636	1520.5	1.44	[GlcN3-NAG4 + H]+
1349.643	1322.0	1.25	[GlcN7-NAG + H]+
1371.615	1234.0	1.17	[GlcN7-NAG + Na]+
1378.681	649.0	0.61	[GlcN2-NAG5 + Na]+
1434.775	1095.0	1.04	[GlcN5-NAG3 + 2H]+
1440.688	2192.0	2.07	[NAG7 + H]+
1462.704	5709.0	5.40	[NAG7 + Na]+
1497.743	2058.0	1.95	[GlcN4-NAG4 + Na]+
1539.717	2258.0	2.14	[GlcN3-NAG5 + Na]+
1581.7	927.0	0.88	[GlcN2-NAG6 + Na]+
1623.741	3304.0	3.13	[GlcN-NAG7 + Na]+
1638.731	2149.0	2.03	[GlcN5-NAG4 + 3H]+
1643.66	1749.0	1.66	[NAG8 + H]+
1665.737	2040.5	1.93	[NAG8 + Na]+
1723.74	5104.0	4.83	[GlcN4-NAG5 + 2Na]+
1846.813	846.5	0.80	[NAG9 + H]+
1861.808	727.5	0.69	[GlcN5-NAG5 + Na]+

1923.869	647.0	0.61	[GlcN3-NAG7 + H]+
1926.852	653.5	0.62	[GlcN4-NAG6 + 2Na]+
2042.902	2174.5	2.06	[GlcN5-NAG6 + H]+

Chit33-ChBD

m/z	Intensity (counts)	% Intensity	Identified COS
222.093	845.0	5.43	[NAG + H]+
425.237	495.5	3.18	[NAG2 + H]+
586.315	231.0	1.48	[GlcN-NAG2 + H]+
628.335	1001.5	6.43	[NAG3 + H]+
789.403	878.0	5.64	[GlcN-NAG3 + H]+
811.379	259.0	1.66	[GlcN-NAG3 + Na]+
831.42	4928.0	31.64	[NAG4 + H]+
853.394	6414.0	41.18	[NAG4 + Na]+
992.469	261.0	1.68	[GlcN-NAG4 + H]+
1014.443	173.0	1.11	[GlcN-NAG4 + Na]+
1195.557	89.0	0.57	[GlcN-NAG5 + H]+

Data obtained in optimal pH and temperature conditions for the indicated Chit33 variants. $[M+H]^+$ and $[M+Na]^+$ peaks were detected in positive mode. Only main peaks in the referred mass interval were indicated. Relative percentage based on "Intensity (counts)".

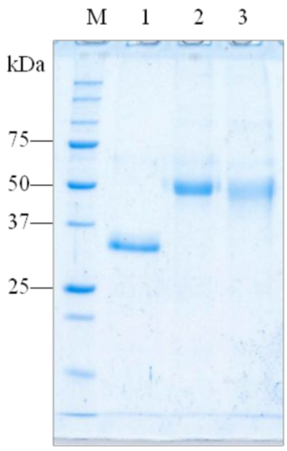

Figure S6. SDS-PAGE analysis of the concentrated Chit33 variants previously expressed in *P. pastoris*. Chit33 (1), Chit33-CBD (2) and Chit33-ChBD (3). Samples were 10-times concentrated after 5 days of the corresponding protein variant expression. Two µL of each sample were loaded. Numbers on the left of each panel indicate the positions of molecular mass standards (lane M) in kDa.

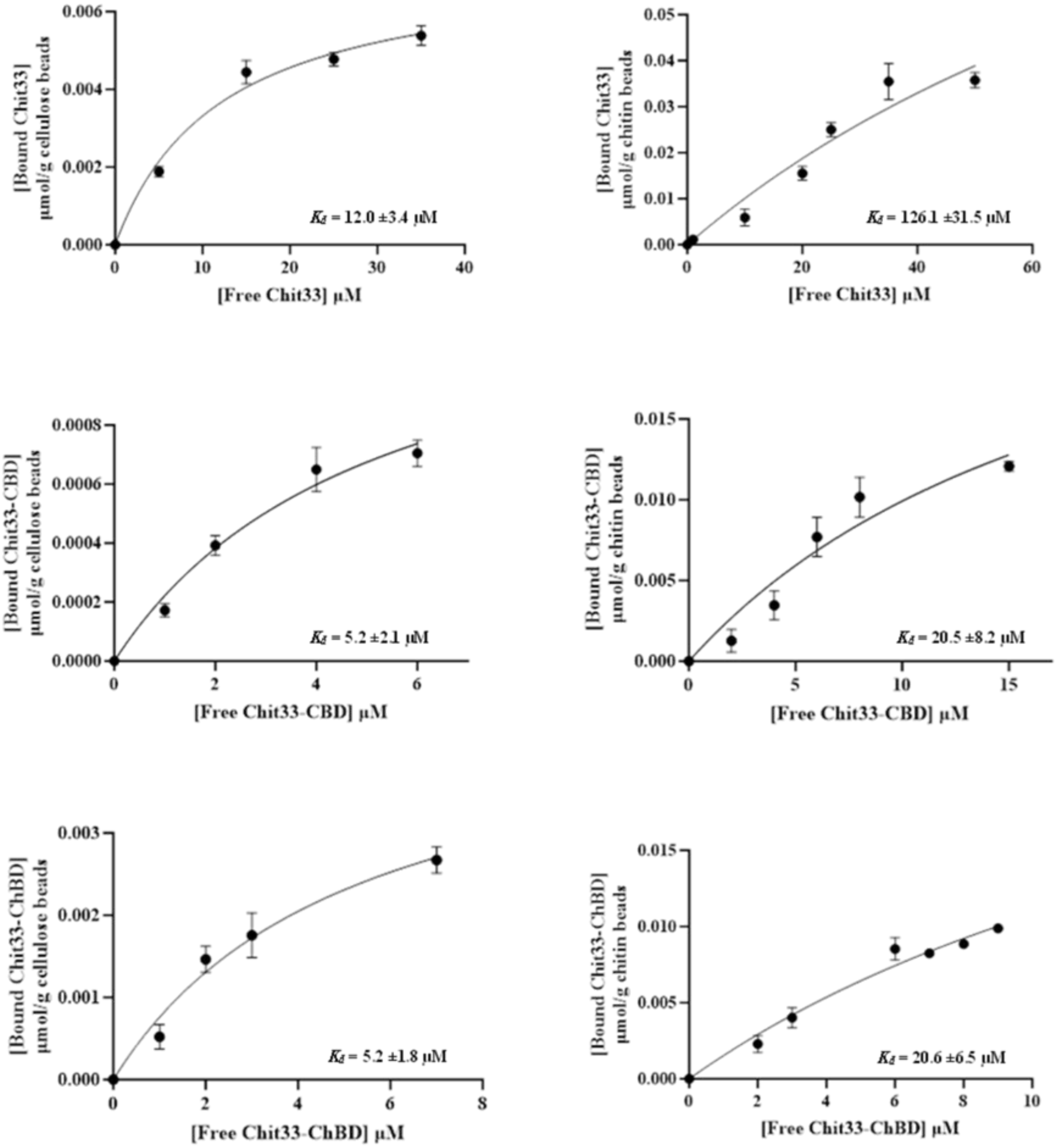

Figure S7. Dissociation constants (K_d) fits for the binding of Chit33 protein variants to cellulose and chitin beads. Reaction assays (125 µL) containing 10 mg of beads (cellulose or chitin) and 1-50 µM of the protein variants in Binding Buffer were incubated at 4°C for 2 hours using a revolver rotator (Labnet Inc., USA) at 20 rpm. Protein concentrations before and after incubation, were measured spectrophotometrically (A_{280}; extinction coefficients predicted with Expasy ProtParam) to estimate bound protein. This data was fitted via nonlinear regression using GraphPad Prism 10.0. Assays were performed in triplicate with blanks (Binding Buffer + beads). The different protein variant-bead combination is indicated in the Y axis of each graph.
